# Assessing Bandung's Governance Challenges of Water, Waste, and Climate Change: Lessons from Urban Indonesia

**DOI:** 10.1002/ieam.4334

**Published:** 2020-10-06

**Authors:** Annisa N Rahmasary, Steven HA Koop, Cornelis J van Leeuwen

**Affiliations:** ^1^ KWR Water Research Institute Nieuwegein the Netherlands; ^2^ Copernicus Institute of Sustainable Development Utrecht University Utrecht the Netherlands

**Keywords:** Bandung City, Water pollution, Water governance, Waste management, Climate change

## Abstract

This study assesses the gaps, opportunities, and priorities of Bandung in managing its water and waste challenges. The City Blueprint Approach is used to identify pressures, to measure the city's Integrated Water Resources Management performance, and to assess its governance. Based on the analyses of Bandung, 4 topics are discussed in more detail: 1) the transferability of the lessons from Bandung, 2) the challenges of solid waste management in Indonesian cities, 3) community‐based sanitation, and 4) implications for informal settlements. The assessment reveals that Bandung's basic water services are largely met but flood risks are high and wastewater treatment is poorly covered, leading to large‐scale pollution. This is amplified by extensive land‐use change and poor solid waste collection and treatment, as waste is almost completely dumped in landfills. Proper solid waste handling will reduce landfill dependency. Slum areas are disproportionately affected by climate‐related hazards and continuously under recognized in the discussion of cities' risk and vulnerability, while its dwellers are the most vulnerable members of the society. Bandung has started with slum area legalization which provides slum dwellers with legal security that protects their right to live as well as access to basic public infrastructures. Inadequate monitoring and uncoordinated financial source allocations are among the governance gaps. Governance is reactive and community involvement is low. Yet, Bandung exhibits the characteristics of a collaborative city with the potential to maximize its cross‐stakeholder learning with supportive leadership. Bandung and other cities in Indonesia face multilevel governance gaps. Bandung is recommended to expand the cooperation of private, civil, and public actors and implement network governance and decentralized management approaches focusing on improving the implementing capacity, better monitoring, cocreation, and better exploration of the options for financial support. *Integr Environ Assess Manag* 2021;17:434–444. © 2020 The Authors. *Integrated Environmental Assessment and Management* published by Wiley Periodicals LLC on behalf of Society of Environmental Toxicology & Chemistry (SETAC)

## KEY POINTS

- The City Blueprint Approach is used to identify the pressures of the city of Bandung (Indonesia), to measure the city's Integrated Water Resources Management performance, and to assess its governance.
- High socioeconomic and environmental pressures in Bandung coincide with high urbanization rates, low education rates, high flood risks, high levels of air pollution, and water scarcity.
- Bandung, as many other rapidly growing Indonesian cities, faces multilevel governance challenges, and the improvement of Bandung's governance capacities is the first and most important step.
- Bandung is recommended to expand the cooperation of private, civil, and public actors, and to implement network governance and decentralized management approaches focusing on improving the implementing capacity, better monitoring, cocreation, and better exploration of the options for financial support.

## INTRODUCTION

The urban development in Indonesia and predominantly Java has progressed rapidly as can be observed in the 200 km urban belt between Jakarta and Bandung (Firman 2009). Pressing issues arise from basic needs, for example, housing, water, food, education, and occupation (Koop and van Leeuwen 2017). This often results in urban stress, including the expansion of slum areas in cities that are deprived of basic services, such as improved water supply, sanitation facilities, sufficient living space, durable housing, and secure tenure (Firman 1999). Urban planning requires the integrated management of various sectorial challenges (Philip et al. 2011; Koop and van Leeuwen 2017; Hoekstra et al. 2018), including Integrated Water Resources Management (IWRM). These water‐related challenges (water supply, sanitation, and stormwater management) as well as solid waste collection and treatment are substantially amplified by climate change. Seasonal flooding, drinking water insecurity, and inappropriate management of solid waste and wastewater are commonly found in Indonesian cities in parallel to rapid urbanization (Douglass 2010; Tarigan et al. 2016). The Bandung Basin has experienced extensive land‐use change, in particular, in the northern part of the Bandung region (Sugandi et al. 2017; Pravitasari et al. 2018). This puts even more pressure on the already confined governing capabilities of local governments as cities are expected to adapt to changing circumstances and risks (Storch and Downes 2011; Schreurs et al. 2017; Hoekstra et al. 2018; Kim et al. 2018).

Despite an annual precipitation of 1700 mm (Abidin et al. 2013), the city struggles with water scarcity, pollution, and groundwater depletion (Taufiq et al. 2018). Based on the projections of water demand, Hasbiah and Kurniasih (2019) conclude that Bandung's water demand will exceed its supply by 2034. Bandung covers only limited wastewater treatment with its centralized system, the Bojongsoang Wastewater Treatment Plant (WWTP) in the Bandung Regency. The conditions are worse in informal settlements where more than 121 000 people are residing (Tarigan et al. 2016). A similar pattern applies to other large Indonesian cities since the monetary crisis in the 1990s which caused urban poverty and led to the decreasing ability of the city government to accommodate public services and infrastructures (Firman 1999). In fact, the governance of water‐related challenges is increasingly being recognized as a main priority for becoming climate proof and for achieving the Sustainable Development Goals (SDGs) (Ostrom 2007; OECD 2015; UN Environment 2018). The multilevel governance gaps as published by the OECD (2015) need to be addressed to ensure that the institutional frameworks are in place to be “fit to fix the pipes” (Romano and Akhmouch 2019).

Because the challenges of water governance transcend administrative boundaries and include many different organizations—each with different responsibilities and interests—it is imperative to provide integrated and problem‐oriented approaches instead of single institutions or policies (Berkes 2009; Koop and van Leeuwen 2017; UN Environment 2018). Therefore, this problem‐solving capacity is considered essential for the improvement of water management and governance in cities (OECD 2015, 2016).

The aim of this paper is to identify the main gaps, opportunities and priorities for the city of Bandung to address its challenges of water, waste, and climate change. The focus of this paper is on water governance. The main results are presented to reflect on solid waste and wastewater management in Bandung. This paper is concluded with the gaps, opportunities, and priorities to improve the water governance capacity in Bandung and other Indonesian cities.

## METHODS

**Figure 1:**
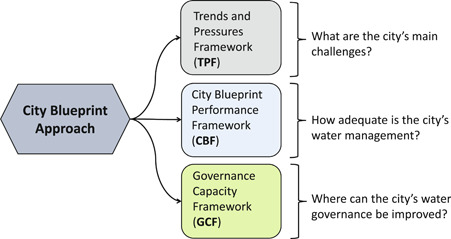
Overview of the City Blueprint® Approach which consists of 3 complementary diagnostic assessment frameworks (Koop and van Leeuwen 2015a, 2015b; Koop et al. 2017).

This study applied the City Blueprint® Approach (CBA) which consists of 3 complementary assessment frameworks (Figure 1). It is a first step to integrate critical challenges into a strategic urban water management approach. Based on this first step, local stakeholders may decide to follow an iterative process to set goals; develop a strategy by exploring different future scenarios; formulate and implement action plans; and monitor, evaluate, and improve the previous steps (Philip et al. 2011; Koop and van Leeuwen 2017).

The CBA has been critically reviewed (Koop and van Leeuwen 2015a, 2015b), leading to a Trends and Pressures Framework (TPF) and a City Blueprint Performance Framework (CBF). The importance of water governance has resulted in the development and application of a third framework: the Governance Capacity Framework (GCF) (Koop et al. 2017). In 2017, the European Commission launched the Urban Water Atlas for Europe—developed around 45 City Blueprint in an accessible and attractive format (Gawlik et al. 2017). Recently, results of the CBA implementation in some Asian cities were published (Rahmasary et al. 2019), and even more recently a major review has been published about the application of the CBA in 32 cities in China (Chang et al. 2020). To date, more than 120 cities in more than 50 different countries have been assessed with the CBA.

The TPF is a quantitative approach and is composed of 24 descriptive indicators divided over 4 categories (social, environmental, financial, and governance). Indicators are scored on a scale from 0–10, where 0 means no concern and 10 is high concern. Data for most of the TPF indicators are national and can be found in open access websites such as the CIA, World Bank, OECD, and WHO (Koop and van Leeuwen 2020a). Only 1 minor revision has been applied in the CBF. One indicator of the CBF has been deleted (Public participation), and the category descriptions have been modified. The 24 indicators of CBF are scored quantitatively mostly using municipal data on a scale from 0 (low performance) to 10 (high performance) and their geometric mean is known as the Blue City Index (BCI) (Koop and van Leeuwen 2020b).

The GCF is a diagnostic semiquantitative assessment consisting of 9 enabling governance conditions that together determine how well governmental and nongovernmental stakeholders cooperate and what their joint problem‐solving governance capacity is to address water challenges (Koop et al. 2017). A total of 9 conditions are distinguished, each with 3 indicators that are scored according to a Likert scale ranging from very encouraging (++) to very limiting (−−) the overall governance capacity to address each challenge (Koop and van Leeuwen 2020c). Bandung's water governance is being assessed with respect to 5 challenges: 1) flood risk, 2) drinking water supply, 3) Municipal Solid Waste (MSW) collection and treatment, 4) wastewater treatment, and 5) urban heat islands (UHI) effects.

The CBA is regularly updated in a learning‐by‐doing fashion with input from stakeholders. Details of the updated TPF and CBF can be found in the Supplemental Data.

## RESULTS

### Bandung's social, environmental, financial, and governance challenges according to the TPF

**Figure 2:**
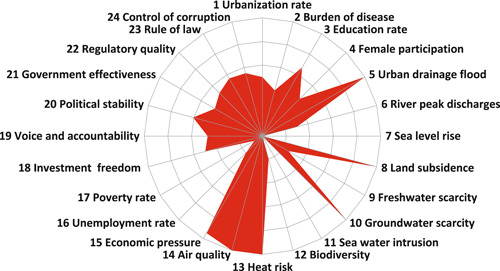
Results of the TPF of Bandung. The indicators are scored on a scale between 0 (center of the circle; no pressure at all) to 10 (periphery of the circle; very‐high pressure).

Bandung faces high social, environmental, financial, and governance pressures. Urban drainage flooding, land subsidence, groundwater scarcity, heat risk, air pollution, and economic pressure are major challenges (Figure 2). Bandung is located on a highland where most of its areas are upstream of the river basin. However, there is a high pressure of urban drainage flooding because blue and green areas cover less than 13% of urban Bandung (Diskamtam 2015). There is increasing urban expansion in the northern area of Bandung which has higher elevation and is supposed to retain water from precipitation events (Pravitasari et al. 2018). This condition is worsened by frequent flash floods from intense rainfall that cannot be accommodated by the city's drainage (Tarigan et al. 2016) and by land subsidence (Figure 2) which occurs at an average rate of 8 cm/y (Abidin et al. 2013). Bandung and many other cities in Indonesia are vulnerable to climate change as a result of increased temperatures and extreme weather events, such as longer periods of heat waves, droughts, and heavy rains (USAID 2020).

Furthermore, the average percentage of children completing their primary education is only 93.5% (UNICEF 2018). Tertiary education normally requires, as a minimum condition of admission, the successful completion of education at the secondary level and is 36% in Indonesia (Figure 2).

People in cities are attracted by the high opportunity to alleviate their economic stability, but the significant disparities that exist often drive them into impoverishment (Firman 1999; Winarso 2010). The social and financial pressures of Bandung stem from the ongoing urbanization driven by its high economic activities. Indonesia has an average annual urbanization rate of 2.27% (CIA 2020). This contributes significantly to the expansion of the slum population in Bandung, where around 30 281 slum households are scattered over more than one‐half of Bandung's districts (Tarigan et al. 2016). Meanwhile, Indonesia's average gross domestic product per capita is US$4391year (IMF 2017), which implies a large economic pressure.

### City Blueprint assessment of Bandung's integrated water resources management

**Figure 3:**
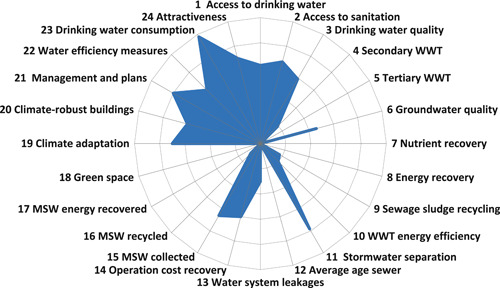
The City Blueprint of Bandung based on 24 performance indicators. The indicators are scored on a scale between 0 (center of the circle; very poor performance) to 10 (periphery of the circle; excellent performance).

The City Blueprint indicator scores are shown in Figure 3, and the BCI of Bandung is 2.6, categorizing Bandung as a “wasteful city” (Koop and van Leeuwen 2015b). Indicator 14 (operating cost recovery of water and sanitation services) and indicator 24 (attractiveness) scored high. Bandung's scores for access to sanitation and drinking water are 6.2 and 6.7, respectively, while drinking water quality received a score of 5.9, showing that there is room for improvement. Green space in Bandung is very low and only 12.1% (Diskamtam 2015). Municipal Solid Waste is another challenge for Bandung. The city can improve on solid waste recycling and energy recovery (indicators 16 and 17). The same holds for wastewater treatment, water infrastructure, and green space.

A centralized system that connects wastewater from houses to the WWTP is difficult to implement in the city due to the lack of available land and skilled manpower, next to the high‐cost and high‐energy requirements (Hendrawan et al. 2013). Bandung's Bojongsoang WWTP uses stabilization ponds and connects around 35% of the population via sewerage networks (Hendrawan et al. 2013). However, its performance is deteriorating due to insufficient maintenance and overflow from nondomestic wastewater (Prihandrijanti and Firdayati 2011; WW02). Instead, septic tanks are widely used by more than 83% of Bandung's population as storage and containment facilities for wastewater from the toilets, that is, fecal sludge (Prihandrijanti and Firdayati 2011; Nastiti et al. 2017). The rest of the untreated domestic wastewater is discharged via an open drainage channel into the rivers or even directly into the environment (Prihandrijanti and Firdayati 2011). The conditions worsen in informal settlements where connection to the central WWTP is nonexistent. Among the slum population in the Greater Bandung Area, only 16.8% collect their wastewater into septic tanks, and 43.4% dispose their wastewater into drainage networks, while 39.6% discharge wastewater directly into rivers (Sofyan et al. 2016).

Tirtawening, the water utility in Bandung, covers about 73% of its population (Hasbiah and Kurniasih 2019) by treating surface water and deep (confined) groundwater (Bappeda 2014). The water system leakage of 35% is substantial (Indicator 13) and is a result of both physical and nonaccounted losses, also known as nonrevenue water (NRW) from poor performing devices, water theft, low meter accuracy, and errors in meter reading (Moersidik et al. 2015). Besides Tirtawening's piped water, Bandung citizens often utilize multiple water sources, such as borehole, protected dug well, or bottled and refilled water (Nastiti et al. 2013). With the rising demand for drinking water consumption, the city needs to consider demand management and NRW management, next to alternative water supply sources. The current main water sources are river water in the Citarum basin, which is infamously polluted, and groundwater, which has dropped 20–100 m between 1980 and 2004 (Abidin et al. 2013). A large percentage of groundwater extraction is attributed to the industrial sector in the Bandung basin and less than 10% to the domestic water supply (Wangsaatmaja et al. 2006). The domestic use of groundwater comes from shallow wells (<40 m below the surface) by local people, and deep wells (40–250 m below the surface) by the water company (Abidin et al. 2013).

Municipal Solid Waste in Bandung is dominated by organic “wet” waste (Damanhuri et al. 2009; BECA 2014). The production of organic waste increases over the years, and it was recorded as 63.4% of the total MSW in 2012 (BECA 2014). MSW is largely collected and transported to landfills, away from the citizen's sight, which is pertinent to the city's effort to increase its attractiveness. The coverage of MSW collection has improved substantially to around 77% in 2012 (BECA 2014). The residents and informal ectors recycle material (6.58%), compost organic waste (0.45%), and apply open‐burning practices (4.49%) (Damanhuri et al. 2009).

Simultaneously, the local government promotes more green spaces in the city with the intention of coping with climate change. However, with a share of only 12.1% of green space in Bandung (Diskamtam 2015), the city does not comply with standards set out by the Indonesia Spatial Planning Act No 26/2007 (ISPA 2007) that requires a minimum of 30%. Bandung has added more urban parks as public space, yet this was done with a focus on ecosystem services that contribute to the city's aesthetic and social value, not to absorb excess rainwater to alleviate storm events (UH02). The flood sensitivity is worsened by the combined sewer system with limited drainage capacity that covers a very limited area of the city and, given its average age of 20–35 y, is becoming obsolete (Prihandrijanti and Firdayati 2011).

### Bandung's water governance capacity

In general, Bandung can be characterized as a collaborative city, since its cross‐stakeholder learning (indicator 3.3), visionary agents or leadership (indicator 6.3), and clear division of responsibilities (indicator 7.2) are encouraging the overall capacity. Bandung is home to a diverse group of social‐environmental communities such as Gerakan Indonesia Diet Kantong Plastik (Indonesia Plastic Bag Diet Movement), Yayasan Pengembangan Biosains dan Bioteknologi (Bioscience and Biotechnology Development Foundation), Sahabat Kota (Friends of the City) (Suharko 2015; Ferdinand and Fam 2019).

Bandung's overall governance capacity is clearly limited, as shown by 3 indicators (Table 1). Underperformance is observed for smart monitoring (indicator 3.1), which may limit policy evaluation (indicator 3.2) and results in limited statutory compliance (indicator 9.2). Accountable monitoring of the amount of waste brought in and out of the landfill is currently lacking so that it detains any opportunity for research and technology development (SW03). Similarly, the domestic water cycle is hard to monitor, particularly with water system leakages (WS02) and the common practice of wastewater disposal to rivers (WW01). The well‐developed stakeholder engagement process (condition 4), continued financial security (indicator 8.2), and adequate use of policy instruments (indicator 9.1) are sometimes hampered by the absence of reliable monitoring data (indicator 3.1). Bandung's water management network provides only limited freedom for individuals to develop new alternatives and innovative approaches (indicator 7.1). As a consequence, the role of entrepreneurial agents of change—individuals that gain access to resources, seek and seize opportunities, and contribute to the decision‐making process—is somewhat inhibited (indicator 6.1).

**Table 1:** Summary of governance capacity indicator scores for Bandung. Indicator scores differ from very encouraging (++), encouraging (+), indifferent (0), limiting (−), and very limiting (−−) the overall capacity to address each of the 5 identified water‐related challenges

Conditions	Indicators	Water challenges				
		Flood risk	Water scarcity	Solid waste treatment	Wastewater treatment	UHI
1. Awareness	1.1 Community knowledge	0	0	+	0	−
	1.2 Local sense of urgency	0	0	+	0	−
	1.3 Behavioral internalization	0	−	+	−	−
2. Useful knowledge	2.1 Information availability	+	0	0	0	−
	2.2 Information transparency	+	−	0	0	−
	2.3 Knowledge cohesion	0	0	0	0	0
3. Continuous learning	3.1 Smart monitoring	−	−	−	−	−
	3.2 Evaluation	−	−	−	−	−
	3.3 Cross‐stakeholder learning	+	+	+	0	+
4. Stakeholder engagement process	4.1 Stakeholder inclusiveness	0	0	+	+	0
	4.2 Protection of core values	−	0	+	+	0
	4.3 Progress and variety of options	0	0	+	+	−
5. Management ambition	5.1 Ambitious and realistic management	0	0	+	0	0
	5.2 Discourse embedding	0	0	0	0	0
	5.3 Management cohesion	0	+	+	0	0
6. Agents of change	6.1 Entrepreneurial agents	−	0	0	+	0
	6.2 Collaborative agents	0	0	+	+	0
	6.3 Visionary agents	0	+	+	+	+
7. Multilevel network potential	7.1 Room to maneuver	−	0	0	0	0
	7.2 Clear division of responsibilities	+	+	+	+	+
	7.3 Authority	0	0	+	0	−
8. Financial viability	8.1 Affordability	0	0	0	0	0
	8.2 Consumer willingness to pay	0	0	0	0	−
	8.3 Financial continuation	−	0	0	0	0
9. Implementing capacity	9.1 Policy instruments	0	0	0	0	0
	9.2 Statutory compliance	−	−	−	−	−
	9.3 Preparedness	0	0	0	−	−

The governance capacity to address MSW is relatively well developed. In particular, awareness (condition 1), stakeholder engagement (condition 4), management ambition (condition 5), and network potential (condition 7) are established. Moreover, collaborative agents and visionary agents (indicators 6.2 and 6.3) are actively bringing stakeholders together and pushing for sustainable long‐term strategies to tackle issues of solid waste management (SW01;04). This well‐developed capacity may be the response to the substantial solid waste challenges that Bandung has faced in previous decades. In particular with respect to flood challenges, it was found that stakeholders do not feel confident that their core values—flood security for their properties—would not be harmed during their engagement in local decision making (indicator 4.2; FL03; UH03). If left unaddressed, this gap where people are often unaware of or even excluded means that the government officials will be perpetually lacking the capacity to enforce the regulations and promote the policies (Fulazzaky 2014). Water managers of Bandung have prioritized access to sanitation and wastewater treatment coverage. The main objective is to promote behavioral change (indicator 1.3) among the citizens through community‐based sanitation programs (WW01;04). This approach necessitates high levels of community knowledge (indicator 1.1) and a local sense of urgency (indicator 1.2). The latter can be considered as a key challenge for the city, in particular in its slum areas (WW03). There is a need of a better understanding of adverse behavior, risk perception, and risk management strategies among these vulnerable households (Nastiti et al. 2017).

With respect to urban heat islands, a general lack of knowledge, awareness, or comprehensive policy (conditions 1, 2, and 5) indicates that the issue has a low political priority and is merely viable through the integration of heat adaptation measures with other urban strategic goals such as the development of green space to combat air pollution (UH02), flood alleviation (UH01), and the improvement of living conditions in the city (UH03).

## DISCUSSION

### Transferring the results of Bandung to other cities

The GCF analysis (Table 1) shows that Bandung faces multilevel governance gaps. Bandung's underperformance is clearly observed for “smart monitoring” (indicator 3.1) which may limit “policy evaluation” (indicator 3.2) and results in limited “statutory compliance” (indicator 9.2). This is in line with the conclusions of Sholeh et al. (2019) who conclude that Bandung fails to implement 3 instruments of policy innovation as part of the smart city policy in Bandung, that is, by regulation, economic and financial instruments and soft instruments. The findings are confirmed by other studies (Antasari 2017; Nastiti et al. 2018; Taufiq 2018; Wang et al. 2018; Hasbiah and Kurniasih 2019; OECD 2019a). Koop and van Leeuwen (2015b) demonstrated a high correlation between the BCI and governance effectiveness as defined by the World Bank (2020). Governance capacity is a premise for improved water management performance and Koop (2019) illustrated that statistically by showing high correlations between BCI and both the governance capacity index and the implementing capacity (indicator 9) in 15 cities.

Two cities have been assessed in Indonesia: Bandung and Jakarta. The City Blueprint of Jakarta (Figure 3 in Rahmasary et al. 2019) hardly differs from the City Blueprint of Bandung (Figure 3). Their indicator scores are almost identical. According to Koop and van Leeuwen (2015b), these cities can be categorized as wasteful cities: “Basic water services are largely met but flood risk can be high and wastewater treatment is poorly covered. Often, only primary and a small portion of secondary wastewater treatment is applied, leading to large‐scale pollution. Water consumption and infrastructure leakages are high due to the lack of environmental awareness and infrastructure maintenance. Solid waste production is high, and waste is almost completely dumped in landfills. Governance is reactive, and community involvement is low.” The commonality is that improving IWRM in these cities is not possible without adequate solid waste and wastewater collection and treatment (Koop and van Leeuwen 2017). Other Asian cities, such as Seoul and Singapore, have much higher BCIs and adequately deal with their water and waste challenges (Kim et al. 2018; Rahmasary et al. 2019) as a result of better governance capacities (Kim et al. 2018; Rahmasary et al. 2019).

**Figure 4:**
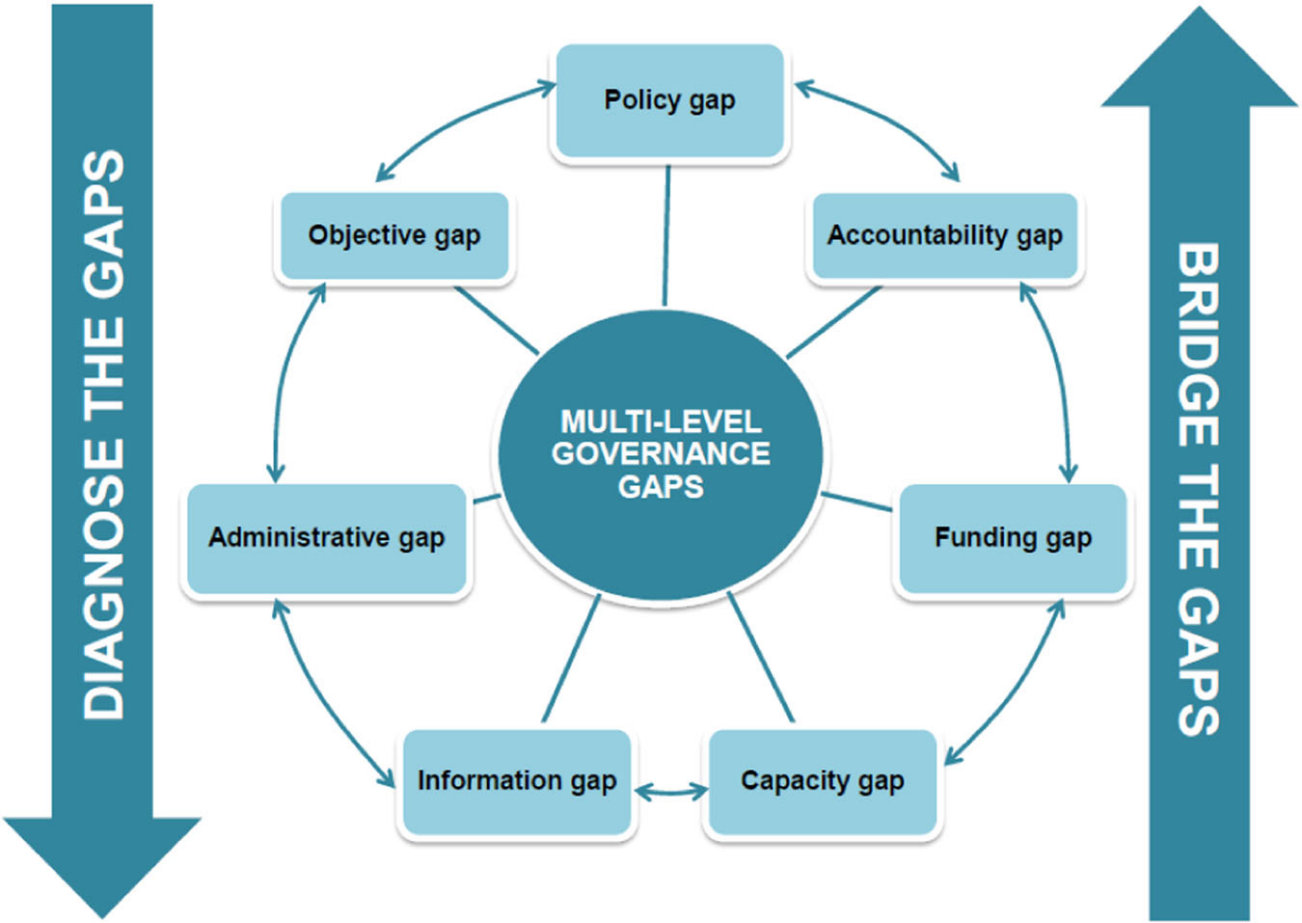
Multilevel governance framework: Mind the gaps, bridge the gaps (OECD 2015).

There are similarities in the causal links leading to the categorization as wasteful cities. The main governance challenges are most likely institutional fragmentation, ambiguous legislation, and poor implementation of multilayered governance, as well as matters such as limited capacity at the local level, unclear allocation of roles and responsibilities, fragmented financial management, and uncertain allocation of resources (OECD 2015, 2016; Figure 4). Long‐term strategic plans and insufficient resources are often lacking to measure performance. This leads to weak accountability and little transparency (Koop and van Leeuwen 2017). Often, these challenges are rooted in inadequately coordinated goals and insufficient steering of the interactions between stakeholders in the water cycle (OECD 2015, 2016).

Bandung can benefit from a long‐term and consistent policy strategy by focusing on the implementation of a network governance model, building on the cooperation of private, civil, and public actors, as well as decentralized management approaches (OECD 2015; Romano and Akhmouch 2019). While the GCF analysis shows that “cross‐stakeholder learning” is present for almost all challenges (Table 1), the available scientific knowledge has not been fully utilized by the local government. The funding of pilot projects is relevant, for example in managing MSW or domestic wastewater, but a long‐term implementation of innovations takes extra effort, as observed from the “indifferent” (0) and “limiting” (−) scores of “progress and variety of options,” “room to maneuver,” and “authority” (indicators 4.3, 7.1 and 7.3), as shown in Table 1.

The measures regarding the 5 water‐related challenges in Bandung are predominantly managed by city‐level government bodies. However, the influence from central government sometimes hampers the ability of local officials to apply innovative measures (Pihkala et al. 2007; Winata et al. 2017). To avoid socio‐institutional inertia, the presence of agents of change in government bodies is required to promote these changes. Likewise, the situation in Surabaya, Bandung uses the “lead‐by‐example” approach with its major goal being up and front in implementing measures (Winata et al. 2017). Nevertheless, improving the city governing capacity by enabling the participation of other agents of change, including the private sector, is preferred and necessary. Intense communication between these stakeholders can provide useful interactions between the knowledge producer and its target groups to enhance the usability of the produced knowledge (Dilling and Lemos 2011; Schreurs et al. 2017; Laeni et al. 2019). Expectedly, the decision making on both “soft” and “hard” measures for the cities will benefit from the cumulative and synergetic effects (Storch and Downes 2011).

Awareness, stakeholder engagement, and agents of chance (Table 1) are needed to bridge the gaps between government and nongovernmental stakeholders. By sharing the results with other cities (city‐to‐city learning) and twinning (institutional networking), one can benefit from both effective practices as well as failures in managing major water challenges (Tortajada 2010; OECD 2015; Koop and van Leeuwen 2017). Many cities in Indonesia, including Bandung, join the Association of Indonesia Municipalities (APEKSI). APEKSI provides roundtable discussions, knowledge fora, and working groups for various strategic issues. However, the information dissemination is not yet effective. Cities' representatives have low participation in the association's program. One of the reasons is that cities are already occupied with the local strategy formulation (Syahidah et al. 2019). In this situation, cities can better prioritize the issues they want to solve (Sholeh et al. 2019) and tailor such fit‐for‐purpose measures in order to address their own urban water challenges.

### The challenges of MSW in Indonesian cities

Indonesia's economic success has come at a high environmental cost. Bandung relies on ecosystem services to maintain its quality where people dispose waste directly into the environment. Bandung produces about 1500 t of solid waste per day (Antasari 2017). With more than one‐half of Bandung's MSW being organic, composting is a better measure to treat solid waste (Damanhuri et al. 2009; Zurbrügg et al. 2012; Antasari 2017). However, the public is reluctant to transfer their organic waste into fertilizer products, as it is a time‐demanding process with limited market opportunities. Bandung also depends on landfilling practices due to its relatively cheap method in the short term. However, it also leads to soil and groundwater pollution that may lead to large overall societal costs (Guerrero et al. 2013; Gupta et al. 2015; Koop and van Leeuwen 2017). To reduce the dependency, the government has issued Government Regulation No. 81/2012 (GR 2012) about solid waste reduction, reuse, and recycling, and the Minister of Environment Regulation No. 13/2012 about waste banks (Lubis 2018). The waste banks promote solid waste separation, reduce‐reuse‐recycle (3R), and zero waste initiatives, since they pay their customers for valuable waste, such as plastic, metal, and paper (Dhokhikah et al. 2015). The city even promotes using a mascot, for example, “Kang Pisman” that is an acronym of “Kurangi” (reduce), “Pisahkan” (separate), and “Manfaatkan” (reuse) and is personified as a Sundanese man wearing traditional clothing (Wang et al. 2018; Ferdinand and Fam 2019).

Although there is a growing awareness, changing the public attitude will take a long time and explains why the present focus is still on improving collection and transport to landfills. It also requires the city government to attract financial support for installing and operating city‐level composting or recycling units. Bandung has no solid‐waste tax, but the citizens pay a very cheap fee for solid‐waste services. Many people also pay the informal sector to help with waste collection and transportation (BECA 2014; SW03). Current initiatives for recycling and composting are either initiated and controlled by government or by partnerships (Damanhuri et al. 2009). These incentives are not seen as attractive, but cooperation with the private sector to increase financial attractiveness, and thus stimulating public participation, is a viable option. Examples are the city's cleanliness agency of Surabaya and Unilever Indonesia who collaborate for the Green and Clean program (Dhokhikah et al. 2015; Winata et al. 2017) and the Gianyar Waste Recovery Project in Bali (Zurbrügg et al. 2012).

Meanwhile, wastewater management requires a greater effort since people are generally unaware of the relationship between proper wastewater treatment and their health (Nastiti et al. 2013). While people believe the government should take care of their wastewater affair, the government struggles with the system as well. Building sewerage infrastructure to connect houses to wastewater networks is too capital‐intensive to be applied throughout urban Indonesia (OECD 2019b). Community‐based sanitation (CBS) requires less extensive infrastructure investments but more societal engagement. In Bandung, the community participants of the CBS program pilot project are consulted and, as a result of this engagement, the sense of ownership of the communal system is considerably high. It increases their involvement in maintenance but does not necessarily empower them (Prihandrijanti and Firdayati 2011; Winata et al. 2017). The evaluation of CBS in Bandung has shown that the majority of users are maintaining the installations, yet they do need to be supervised and assisted. If the government can further promote the practice of CBS, it will gain more public interest and attract private collaboration (Sofyan et al. 2016).

While CBS with active public engagement shows the highest potential to enhance public sanitation, particularly for overcrowded urban settlements, it has comparably low removal efficiencies (Kerstens et al. 2015). With adequate supervision and improvements to ensure the quality and long‐term viability, CBS could alleviate the sanitation status. Furthermore, this could improve Bandung's governance capacity by facilitating meaningful interactions between stakeholders and improving citizens' knowledge about wastewater treatment. Since the city has Bojongsoang WWTP, an optimized combination of existing centralized networks and decentralized systems seems to be the most viable alternative (Prihandrijanti and Firdayati 2011).

### Implications for informal settlements

The highest percentage of populations living in informal settlements (slums) are found in cities that are categorized as cities “lacking basic water services,” followed by “wasteful cities” (Koop and van Leeuwen 2015b). Many cities in Asia, including Bandung, belong to the same category (UN 2018; Rahmasary et al. 2019). Slum areas are disproportionately affected by climate‐related hazards and are continuously underrecognized in the discussion of the cities' risk and vulnerability, while its dwellers are the most vulnerable members of the society (Jamil 2013; Jones 2017; Winata et al. 2017). If a city wishes to address its waste and water challenges, it should give the utmost attention to the slum regions.

Slum management is multidimensional and residents are often cautious and resist change since they depend on the current living situation (Jamil 2013). Combining insights from policy makers and experts with local communities and civil society organizations is pivotal in developing spatial and adaptive measures (Laeni et al. 2019). Bandung has started with “slum area legalization” (Tarigan et al. 2016), which provides slum dwellers with legal security that protects their right to live as well as access to basic public infrastructures. Surabaya takes this approach further by acknowledging slums in its development strategy, not to eliminate their existences but to empower them. The Kampung Improvement Program (KIP) allows locals to detect their priorities and to contribute their resources (e.g., money, labor, and building materials) that results not only in improved basic infrastructure but also in establishing home‐based industries (Winata et al. 2017). Grassroots organizations can improve the process further by offering advocacy and consultation. Their activities usually aim for recognition and support of the improvement or protection of basic services in slum areas (Birch 2016). This is observed in the revitalization of the Cikapundung River's slum area. Some of the adjacent residents agreed to be relocated while the condition of the remaining slum area has been alleviated with the maintenance of the public space “Teras Cikapundung.” The local community, comprising slum residents, are now taking care of the area, especially by supervising solid waste management (FL02), which will also reduce flood risks in this area.

## CONCLUSIONS

High socioeconomic and environmental pressures in Bandung coincide with high urbanization rates, low education rates, high flood risks, high levels of air pollution, and water scarcity. IWRM performance can be improved by focusing on basic water services, particularly wastewater treatment, as well as solid waste collection, recycling, and energy recovery. Options for improvement for Bandung based on cobenefits and prevention of further damage therefore reside in these 2 areas of wastewater and MSW handling and treatment, that is, on 1) supporting CBS practice and 2) reducing landfill dependency.

CBA has provided a baseline diagnosis of the main barriers, enablers, and learning practices. Next steps are required to explore cost‐effective solutions. Bandung, as many other rapidly growing Indonesian cities, face multilevel governance challenges (Figure 4 and Table 1). The improvement of Bandung's governance capacities is the first and most important step. Bandung's implementing capacity can be improved by enforcing environmental legislation and providing more incentives and loans, next to focusing its monitoring systems of key practices to enable effective evaluation for future decisions.

More importantly, Bandung would benefit from the implementation of a network governance model, building on the cooperation of private, civil, and public actors, as well as decentralized management approaches. For example, stakeholder learning and partnerships between the water utility and the research community can map out future water supply plans, while government officials and local communities can cosupervise and assist CBS implementation. In gaining financial support, besides collaborating with the private sector, Bandung can use its status as the province capital city to utilize the additional financial support from regional and national governments. This financial support can be used to develop Bandung's urban infrastructures in a more sustainable manner by safeguarding the future of its inhabitants and resources.

## Disclaimer

The authors declare no conflict of interest.

## Data Availability

Data and associated metadata and calculation tools are available upon request by contacting corresponding author Kees van Leeuwen (Kees.van.Leeuwen@kwrwater.nl).
